# Paranoia and Grandiosity in the General Population: Differential Associations With Putative Causal Factors

**DOI:** 10.3389/fpsyt.2021.668152

**Published:** 2021-04-30

**Authors:** Julia M. Sheffield, Aaron P. Brinen, Daniel Freeman

**Affiliations:** ^1^Department of Psychiatry and Behavioral Sciences, Vanderbilt University Medical Center, Nashville, TN, United States; ^2^Department of Psychiatry, University of Oxford, Oxford, United Kingdom

**Keywords:** paranoia, grandiosity, delusions, worry, cognitive-behavioral approaches

## Abstract

Worry, negative self-beliefs, and sleep disturbance have been identified as contributory factors to the onset, maintenance, and severity of paranoia. We tested the specificity of these contributory factors to paranoia compared to grandiosity, a different type of delusional ideation. Data were used from 814 adults from the Nathan Kline Institute-Rockland (NKI-Rockland) study, a general population dataset. Paranoid and grandiose delusional ideation was assessed using the Peters Delusions Inventory (PDI-21) and correlated with self-reported worry (*n* = 228), negative self-beliefs (*n* = 485), and sleep quality (*n* = 655). Correlations were compared using Fisher's r-to-z transform to examine whether the magnitude of relationships differed by delusion type. Paranoia was significantly associated with worry, negative self-belief, and sleep quality. Grandiosity demonstrated significantly weaker relationships with worry and negative self-beliefs. Relationships with sleep quality were similar. We replicate previous reports that worry, negative self-beliefs and sleep quality are associated with paranoid ideation in the general population. We extend these findings by demonstrating that these contributory factors, particularly worry and negative self-beliefs, are associated with paranoid ideation to a greater extent than grandiosity. This suggests a degree of specificity of contributory factors to different types of delusional thinking, supporting the pursuit of specific psychological models and treatments for each delusion type.

## Introduction

Developing precise psychological models of mental experiences is critical for advancing treatment. Increasingly, research is focused on leveraging cognitive-behavioral models to identify appropriate treatment targets (1, 2). The study of delusions has benefited from this approach. Meta-analysis indicates that studies targeting specific contributory factors (e.g., self-esteem, worry) may demonstrate greater effects on the improvement of delusion severity than a broader, formulation-driven cognitive-behavioral therapy for psychosis (CBTp) approach (3). Defining appropriate and specific cognitive-behavioral models of psychiatric symptoms is therefore an important step toward effective and individualized treatments.

The threat anticipation model, a targeted cognitive-behavioral model of persecutory delusions, has been proposed in order to inform treatment development (4). This model suggests a number of factors that contribute to persecutory delusion onset and maintenance (5). Worry brings the threat belief to mind and keeps it there, reducing exploration of alternative perspectives and increasing psychological distress. Negative self-beliefs increase feelings of inferiority and vulnerability to harm from others. Sleep disturbance also contributes to persecutory ideation by increasing negative affect, mood dysregulation, and anomalous internal states. Critically, treatments targeting worry, negative self-beliefs and insomnia improve persecutory delusion severity in patients with schizophrenia (5–9), bolstering evidence that they are causal factors of persecutory ideation (10).

Persecutory delusions are the extreme end of a paranoia continuum that describes unfounded ideas that others intend you harm (11). Paranoia is common in the general population (12). Approximately 10% of individuals without a psychotic disorder endorse the belief that others have been trying to harm them or their interests (13) and 10–20% of individuals endorse paranoid thoughts with strong conviction and significant distress (14). Even at sub-clinical levels, paranoia is clinically relevant. Greater endorsement of paranoid thinking has been associated with increased suicidal ideation, greater substance use, poor social functioning, and lower levels of happiness (15). Studying milder variants of paranoia can inform the understanding of clinical disorder. Studying paranoia in the general population has the notable advantage of enabling larger sample sizes, which can, for example, provide the power to test differential associations.

Paranoia is one type of delusion; another is grandiosity. Grandiose ideation reflects the belief that one has special powers, abilities, or purpose. Grandiosity is present in the general population, although prevalence rates vary widely based on sample (8–65%) (16, 17). Delusion types, such as paranoia and grandiosity, will share contributory factors, but they are also expected to have associated features that set them apart (17, 18). Twin studies, for example, report small to moderate heritability of grandiosity and paranoia separately (19, 20), suggesting biological antecedants of these delusion types that may put individuals at differential risk for one delusion vs. the other. Environmental factors may also specifically contribute to paranoia vs. grandiosity, such as exposure to childhood trauma (21). Psychological processes are also differentially associated with persecutory and grandiose delusions (22). For instance, longitudinal analysis of psychotic symptoms demonstrated poor impulse control activates grandiosity, whereas anxiety symptoms activate paranoia (23).

Despite their differences, persecutory, and grandiose delusions tend to co-occur in schizophrenia (24) and in non-clinical samples, grandiose, and paranoid ideation are correlated (25). This overlap signals shared psychological mechanisms, motivating the identification of common and unique features for more precise treatment development. In fact, prior accounts of grandiosity point to factors that are core to the cognitive-behavioral model of paranoia as contributing to grandiosity, making grandiosity an ideal candidate for examining specificity. It has been suggested, for instance, that grandiosity plays a role in the development of paranoia in some individuals, fueled by worry that one will be targeted for their special gifts (26). Negative self-beliefs also play a potentially interesting role in grandiosity. Although seemingly counter-intuitive, the “delusion as defense” model suggests that grandiose beliefs serve to protect feelings of worthlessness and vulnerability (27). Furthermore, insomnia is commonly reported as a precursor to the development of grandiose beliefs (28). While theoretically compelling, the contribution of worry, negative self-beliefs, and sleep quality to grandiosity requires continued study (22). Directly comparing the contribution of these factors to paranoia vs. grandiosity will help determine their relative specificity.

The current study seeks to address the following aims within a non-clinical general population sample: (1) replicate the associations between worry, negative self-beliefs and sleep to paranoid ideation in a general population sample, (2) determine whether these factors are also associated with grandiosity or are specific to paranoia.

## Methods

### Participants

Participant data were collected as part of the Nathan Kline Institute-Rockland Sample (NKI-Rockland), a large (>1,000 individuals) community-ascertained sample of individuals spanning ages 6–85 (29). Age, ethnicity, and socioeconomic status of the sample is representative of Rockland, NY, which resembles those of the United States more broadly according to the 2010 U.S. census. The NKI-Rockland sample is a publicly available dataset comprised of self-report, neuroimaging, and genetics data. Participants included in the current analyses were labeled in the NKI-Rockland database as having participated in one of the following sub-studies: (1) Discovery Science, which is the original and primary NKI-Rockland study that includes self-report, neuroimaging, and genetics as well as tests of physiology across the lifespan, (2) Neurofeedback, a sub-study that involves special neuroimaging procedures to examine functional brain networks, or (3) Adult Longitudinal, a sub-study that involves longitudinal neuroimaging of individuals collected within the NKI-Rockland sample. Adult (18+) participants from the NKI-Rockland sample who completed self-report questionnaires measuring delusional ideation, worry, sleep, and negative self-beliefs (described in detail below) were identified from the larger dataset and included in this study (Table 1). These cohorts were partially overlapping. Demographics for these cohorts skewed slightly more female than the U.S. general population (~62% female compared to 51% general population). A Structured Clinical Interview of the DSM-IV-TR (SCID) was conducted on the majority of study participants (86%) and diagnostic data on study participants are presented in Table 1. Across the sample of individuals with available delusional ideation data (*n* = 814), only five individuals had a diagnosis of a psychotic disorder (0.6% of the sample).

**Table 1:** Participant characteristics.

	**PDI-sleep cohort** **(*N* = 655)**	**PDI-negative self-beliefs cohort** **(*N* = 439)**	**PDI-worry cohort** **(*N* = 228)** *70 with diagnostic data
Age [mean (SD); range]	47.93 (19.25)	37.49 (13.70)	39.56 (15.0)
	18–85	18–59	20–70
Sex (% female)	62%	61%	66%
Race (AA/W/O) (% white)	107/490/58 (75%)	97/292 (67%)	33/176/18 (77%)
**Diagnostic data**			
Major depressive disorder	98	77	13
Bipolar disorder	4	4	0
Psychotic disorder	5	2	0
Substance use disorder, past	251	211	36
Substance use disorder, current	20	19	2
Anxiety disorder	66	53	9
PTSD	17	15	3

*Participant characteristics for each cohort with available self-report data. Cohorts are partially overlapping in the following ways: all 439 individuals from the negative self-beliefs cohort are within the sleep cohort. Sixty-nine and 59 individuals from the worry cohort are within the sleep and negative self-beliefs cohorts, respectively. AA, African-American; O, Other race (non-white; non-African-American); PDI, Peters Delusions Inventory; SD, standard deviation; W, White*.

### Study Measures

#### Delusional Ideation

Delusional ideation was measured using the Peters Delusion Inventory-21 (PDI-21). The PDI-21, which assesses delusional ideation, is a valid and reliable measure of delusional thinking in the general population (30). Although the PDI-21 captures a wide survey of delusional ideation, prior factor analysis has identified items consistent with paranoid and grandiose ideation (17, 18). These a-priori factor loadings were used to inform the current study. While the paranoia factor was comprised of four items, we combine here the grandiosity and religiosity factors from prior studies, as grandiosity and religiosity share similar clinical features (31) and these items tap into the belief that one has a special purpose. This also allowed for the measures of paranoia and grandiosity to have the same range of scores, as they both included four items. Scores were calculated as the sum of the item (yes/no), conviction, preoccupation, and distress (range 0–64) as has been done previously (32).

Items comprising the paranoia score were: “do you ever feel as if people seem to drop hints about you or say things with a double meaning?,” “do you ever feel as if some people are not what they seem to be?,” “do you ever feel as if you are being persecuted in some way?,” and “do you ever feel as if there is a conspiracy against you?.” Items comprising the grandiosity score were “do you ever feel as if you are destined to be someone very important?,” “do you ever feel that you are a very special or unusual person?,” “do you feel that you are especially close to God?,” and “do you ever feel as if you have been chosen by God in some way?.”

#### Negative Self-Beliefs

Negative self-beliefs were measured using two scales: the Beck Depression Inventory (BDI-II) (33) and the Achenbach Adult Self-Report (ASR) (34). Items consistent with negative self-beliefs were identified. The BDI-II assesses depressive symptoms over the past 2 weeks on a 4-point scale. The BDI-II consists of two factors: negative cognition and affective-somatic feelings (33). The nine negative cognition items were selected (e.g., self-criticism, self-dislike) consistent with prior reports using these items to measure negative self-beliefs (35). The ASR is a 126-item self-report questionnaire that assesses a range of adaptive functioning problems on a scale of 0 (Not True), 1 (Somewhat or Sometimes True), or 2 (Very True or Often True). Items consistent with negative self-beliefs included six items from the Anxious/Depressed sub-scale (e.g., “I feel that no one loves me,” “I feel worthless or inferior”), and a single item from the Withdrawal sub-scale (“I lack self-confidence”). Within each scale, identified items were first summed, then standardized (*z*-scored) within the sample, and then those standard scores were averaged to obtain a single measure of severity of negative self-beliefs.

#### Sleep

The Pittsburgh Sleep Quality Index (PSQI) is a 10-item self-report questionnaire that assesses usual sleep habits over the past month (36). The PSQI generates seven “component” scores that assess subjective sleep quality, sleep latency, sleep duration, habitual sleep efficiency, sleep disturbances, used of sleeping medication, and daytime dysfunction. A global composite score was used to measure overall sleep quality, which was the sum of the seven component scores.

#### Worry

Worry was assessed using the Penn State Worry Questionnaire (PSWQ) (37) and the Perseverative Thinking Questionnaire (PTQ) (38). Total scores were calculated for each measure (total sum). Relationships with delusional ideation were examined first using the PSWQ and then confirmed with the PTQ. The PSWQ is a 16-item scale that assesses the occurrence, intrusiveness and pervasiveness of an individual's experience with worry, rated from “not at all typical of me” to “very typical of me.” The PTQ is a 15-item scale that measures repetitive negative thinking. The PTQ assesses the nature of these thoughts in terms of intrusiveness, the perceived unproductive nature of the thoughts, and the way the thoughts overtake mental capacity and make it difficult to focus on other things. Perseveration is a core psychological mechanism of worry and previous work has shown correlations between the PTQ, PSWQ, and severity of persecutory delusions in non-affective psychosis (39).

### Statistical Analysis

The NKI-Rockland study is comprised of multiple sub-studies, as well as longitudinal components. Therefore, some questionnaires were administered twice to the same participants, while others were collected as part of distinct sub-studies. If questionnaires were administered twice, the data collection closest to study enrollment was selected for use in the current study. Data on delusions, sleep, and negative self-beliefs were collected as part of the original Discovery Science study visit and were therefore collected during the same study visit for 87% of participants (negative self-beliefs) and 99% of participants (sleep). Worry questionnaires were added to the study later and were collected at one of the following visit types: Discovery Science, Longitudinal, or Neurofeedback, with only 15% completing the measures within the same study visit as delusional ideation. Therefore, “day lag” (time between collection of the questionnaires) is included as a covariate in pairwise correlations. Furthermore, sensitivity analyses were conducted to examine the impact of day lag on the reported findings for worry relationships.

Of the participants who completed the Peters Delusion Inventory-21 (PDI-21), 655 have data on sleep quality, 439 have data on negative self-beliefs, and 228 have data on worry. The overlap amongst these cohorts is as follows: all 439 individuals with negative self-beliefs data also have sleep data, 69 individuals with worry data also have sleep data, 59 individuals with worry data also have negative self-beliefs data, only 56 individuals have data from all self-reports. Across the entire cohort, 814 unique individuals have PDI data.

Analyses were conducted in SPSS v.27. Relationships between delusional ideation types and contributory factors were first examined in parametric pairwise correlations, controlling for age, gender, race, and day lag (for the worry analysis). Magnitude of correlations were then compared statistically using Fisher r-to-z transformation as recommended by Meng et al. (40) for comparing correlated correlations. Sensitivity analysis for the relationships between delusional ideation and worry were conducted to examine the effect of day lag on the correlations. A median split was conducted on the time between completion of the PDI and worry self-reports (day lag) and correlations were conducted within each split-half (without day lag as a covariate). The sensitivity analysis was conducted to further validate the stability of the relationship between worry and paranoia.

Finally, in a supplemental analysis intended to examine the variance explained by all contributory factors to delusional ideation, the 56 individuals with data from all measures were included in a linear regression. Two regressions were conducted (one for paranoia, the other for grandiosity), both including covariates (age, gender, and race), negative self-beliefs, sleep quality, and worry (PSWQ) as independent variables.

## Results

Means scores, ranges, and Cronbach's alpha for each measure are summarized in Table 2. Primary results are presented in Table 3.

**Table 2:** Descriptive statistics for self-report measures.

**Self-report**	**Mean (SD)**	**Range**	**Cronbach's alpha**
PDI paranoia	8.94 (9.03)	0–57	0.76
PDI grandiosity	8.85 (12.63)	0–64	0.84
Negative self-beliefs	1.91 (2.55)	0–15.5	0.90
Worry (PSWQ)	27.91 (11.00)	11–54	0.72
Worry (PTQ)	20.06 (12.24)	0–57	0.96
Sleep quality	8.61 (5.82)	0–31	0.68

*PDI, Peters Delusions Inventory; PSWQ, Penn State Worry Questionnaire; PTQ, Perseverative Thinking Questionnaire*.

**Table 3:** Summary of results.

	**Paranoia**	**Grandiosity**	**Significant difference**
Negative self-beliefs	*r* = 0.28, *p* < 0.001	*r* = 0.09, *p* = 0.07	*z* = 2.92, *p* = 0.004
Sleep	*r* = 0.15, *p* < 0.001	*r* = 0.12, *p* = 0.002	*z* = 0.073, *p* = 0.23
Worry (PSWQ)	*r* = 0.42, *p* < 0.001	*r* = 0.04, *p* = 0.53	*z* = 4.32, *p* < 0.001
Worry (PTQ)	*r* = 0.39, *p* < 0.001	*r* = 0.12, *p* = 0.20	*z* = 3.09, *p* = 0.001

*Correlations between paranoia, grandiosity, and putative causal factors controlling for age, gender, and race. Significant difference in magnitude of correlation coefficients based on Fisher r-to-z transformation. PSWQ, Penn state worry questionnaire; PTQ, Perseverative thinking questionnaire*.

### Negative Self-Beliefs

Self-reported negative self-beliefs were significantly associated with paranoia (*n* = 439, *r* = 0.28, *p* < 0.001) and grandiosity (*n* = 439, *r* = 0.09, *p* = 0.07), indicating that worse negative self-beliefs were associated with worse delusional ideation. The magnitude of the difference in correlations significantly differed (*z* = 2.92, *p* = 0.004), indicating that negative self-beliefs contributed significantly more variance to paranoia than grandiosity.

### Sleep

Sleep disturbance was significantly associated with both paranoia (*n* = 655, *r* = 0.15, *p* < 0.001) and grandiosity (*n* = 655, *r* = 0.12, *p* = 0.002), such that greater sleep disturbance was associated with worse delusional ideation. The magnitude of these relationships did not differ (*z* = 0.73, *p* = 0.23).

### Worry

**Figure 1:**
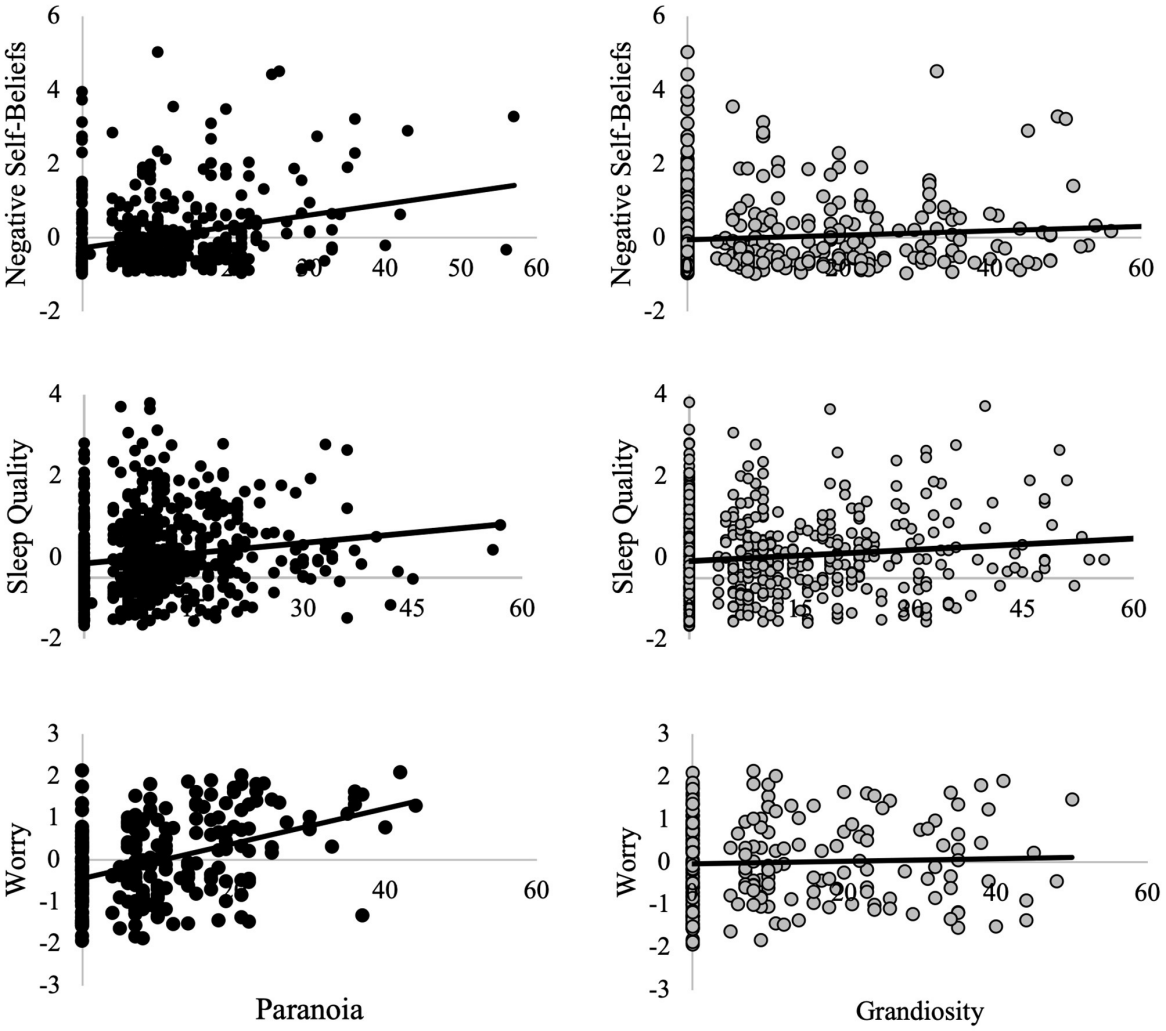
Relationships between paranoia, grandiosity, and contributory factors. Worry, negative self-beliefs, and sleep quality are presented as residuals, controlling for age, sex, race, and the number of days between when the two self-report measures were conducted (“day lag”). Worry was measured throught the Penn State Worry Questionnaire (PSWQ). Negative self-beliefs was measured through the Beck Depression Inventory (BDI-II) and Adult Self-Report Scale (ASR). Sleep quality was measured using the Pittsburgh Sleep Quality Inventory (PSQI). Paranoia and grandiosity were measured with the Peters Delusions Inventory (PDI).

Self-reported worry (PSWQ) was significantly associated with paranoid ideation (*n* = 228, *r* = 0.42, *p* < 0.001) but not grandiosity (*n* = 228, *r* = 0.04, *p* = 0.53; Figure 1). This was a statistically significant difference in magnitude of correlation (*z* = 4.32, *p* < 0.001), indicating a significantly stronger association between worry and paranoia than between worry and grandiosity.

Sensitivity analyses were conducted to examine the impact of day lag on the observed correlations. Median time between collection of the PDI and worry questionnaires was 22 days. For those individuals with ≤22 day lag, paranoia, and worry correlated at *r* = 0.48, *p* < 0.001 whereas grandiosity and worry correlated at *r* = 0.07, *p* = 0.427 (*z* = 3.28, *p* = 0.001). For individuals with >22 day lag, correlation between paranoia and worry was *r* = 0.37, *p* < 0.001 and correlation between grandiosity and worry was *r* = 0.02, *p* = 0.833 (*z* = 2.62, *p* = 0.009). Therefore, stronger associations between worry and paranoid ideation that grandiosity appears to be reliable and not biased by the time between self-reports.

Finally, all the above associations with worry were further validated by examining relationships with the PTQ (*n* = 228). Similar to the PSWQ, perseverative thinking showed a significantly stronger association with paranoia (*r* = 0.39, *p* < 0.001) than grandiosity (*r* = 0.12, *p* = 0.20) (*z* = 3.09, *p* = 0.001). The two worry measures were inter-correlated (*r* = 0.68, *p* < 0.001).

### Sub-sample Regression Analysis

In a smaller sub-sample (*N* = 56) of individuals with all measures completed, covariates and the contributory factors explained 29% of the variance in paranoia [*R*^2^ = 0.29, *F*_(6, 51)_ = 3.47, *p* = 0.006]. Of those variables, only worry was significantly associated with paranoia (β = 0.39, *p* = 0.007). Regarding grandiosity, the contributory factors did not explain significant variance [*R*^2^ = 0.04, *F*_(6, 51)_ = 0.32, *p* = 0.92) and none of the factors were significantly associated with grandiosity.

## Discussion

Negative self-beliefs, sleep disturbance, and worry have been previously identified as contributory factors of paranoia. Here, we examined the specificity of these factors to paranoia vs. grandiosity. We replicated previous findings, demonstrating significant associations between worry, negative self-beliefs, sleep disturbance and paranoia. Worry and negative self-beliefs were significantly more strongly associated with paranoia than grandiosity. Furthermore, in a sub-sample, the contributory factors explained significant variance in paranoia but not grandiosity, suggesting a degree of specificity to paranoia, as opposed to delusional ideation more generally. These data suggest that treatments targeting contributory factors, particularly worry and negative self-beliefs, would not be equally effective in treating all themes of delusional thinking. These findings converge with examination of contributors to persecutory and grandiose delusions in schizophrenia (22), which revealed that depression, anxiety, and negative self-evaluations were positively associated with presence of persecutory delusions but negatively associated with presence of grandiose delusions. The current findings extend this to the general population and reveal that severity of worry and negative self-beliefs are more important for understanding level of paranoid vs. grandiose ideation.

These findings are also interesting in light of a recent qualitative study of grandiose delusions, which identified a number of potential maintenance factors in the experience of grandiosity: meaning-making, anomalous experiences, mania, fantasy elaboration, reasoning biases, and immersive behaviors (28). We note some overlap with maintenance factors of paranoia—for instance sleep disturbances are characteristic of mania, and negative self-beliefs may motivate meaning-making—which are in line with our findings of significant (but relatively less robust) associations between grandiosity, sleep and negative self-beliefs in the current sample; yet the cognitive-behavioral model is relatively distinct from that of paranoia. Furthermore, worry is notably absent from the grandiosity maintenance factors endorsed by patients. Isham et al. (41) suggest that fantasy elaboration may serve a similar cognitive function as worry (i.e., bringing the belief to mind and elaborating on it), but it does not appear to activate the threat-system that is characteristic of worry. Threat anticipation is part of the negative valence system and serves to proactively organize behavior and prepare emotional responses to cope with the impact of potential events. Therefore, interventions targeting threat-systems and negatively-valenced anticipatory feelings may be most effective in treating persecutory beliefs but less useful in treating grandiosity. The current data highlights the important role of using experience-specific models to design interventions, even for experiences under the same “symptom” umbrella (e.g., delusions).

Specificity of contributory factors to paranoia was identified in the context of replicating cross-sectional associations with worry, negative self-beliefs, and sleep quality in a general population sample of American adults. Of these factors, worry demonstrated the strongest relationship with paranoia, explaining 20% of the variance in paranoid ideation and demonstrating a significant relationship with paranoia even when taking the other contributory factors into account. Worry styles in patients with persecutory delusions are similar to those with generalized anxiety disorder (42), reflecting elevated subjective probabilities of future negative events (43) and stronger belief in the likelihood of unpleasant outcomes (44). While it is clear that a relationship between paranoia and worry exists, the directionality of this relationship could not be established in the current study. Prior research using large longitudinal datasets has suggested bidirectionality of anxiety, worry and paranoia (45, 46), suggesting a perpetuating cycle of worry and persecutory ideation. This bidirectionality may further extend to the other contributory factors, for instance worry leading to difficulty sleeping. Given the strength of its contribution to paranoid ideation, treatment of worry in the general population may provide the greatest impact on paranoid thinking, potentially breaking this mutually reinforcing cycle.

Both negative self-beliefs and sleep quality were also significantly associated with paranoid ideation. Negative self-beliefs (e.g., feeling worthless, disliked, and unlovable) make one feel vulnerable to outside threats and are considered central in the hierarchy of paranoia (14). Here, negative self-beliefs were measured in part by the Beck Depression Inventory, reflecting how depressive cognitions may be a risk factor for developing paranoia in the general population, as previously shown (35). Sleep quality, on the other hand, demonstrated a relatively small (although significant) association with paranoia. The small effect size was somewhat surprising given robust associations between insomnia and paranoia identified in epidemiological studies (47–49). The current study used the PSQI, a common measure of sleep quality that assesses a range of sleep quality dimensions, including sleep efficiency and use of sleep medication, which may be less relevant for paranoia. In a pilot clinical trial, treatment of insomnia in schizophrenia significantly improved insomnia at 12 weeks, but not sleep quality as measured by the PSQI (8, 9). Measurement of insomnia specifically is very likely to have revealed more robust relationships with paranoia.

Finally, these findings add to the body of research that implies a shift is needed in the focus of psychosis studies, etiology and treatment, to privilege psychological factors that significantly impact severity of experiences. As opposed to defining psychosis as aberrant and disordered, having a more pragmatic and understandable etiology for delusions can reduce stigma in individuals. Worry, sleep, and self-beliefs are shared human experiences and this study establishes the factors are shared with those in the general population. Treatments have successfully targeted these contributory factors in a patient population and found a positive effect on paranoia (8, 9). Further, patients prefer these factors as targets in treatment (10). Because these factors are less stigmatizing, common amongst, patient and general population, and appealing to patients, they present a useful and potentially effective start point for treatment of individuals with psychosis.

This study is not without limitation. First, is the use of a sample that was not collected with the focused purpose of examining the current questions. One strength of this approach is that we were able to replicate previous findings in an independent, American, general population sample. One downside, however, was that our assessment measures of interest were not consistently completed in the same individuals. This reduced our sample size for some variables and introduced a lag between when some of the measures were completed; however sensitivity analysis and robustness of results with inclusion of day lag as a covariate suggest that these relationships were stable and not meaningfully impacted the time delay. Furthermore, these data are cross-sectional and therefore cannot speak to the development or exacerbation of paranoia or grandiosity and cannot be the basis of any conclusions about causality. Prior work has already shown that worry, negative self-beliefs, and sleep disturbance contribute to the persistence and exacerbation of paranoid thinking in the general population (15). Given the relative strength of associations between worry and paranoia observed in the current study, future studies should examine whether improvement in worry mediates longitudinal changes in sleep, negative self-beliefs and paranoia.

In conclusion, the current study promotes the elucidation of cognitive-behavioral contributors to specific experiences (e.g., paranoia vs. grandiosity) and highlights worry as a particularly important contributor to paranoia. While delusions likely share many broad cognitive and neurobiological alterations (e.g., abnormal predictive coding, reasoning biases) (50, 51), defining experience-specific models will help maximize treatment outcomes across all levels of severity.

## Data Availability Statement

Publicly available datasets were analyzed in this study. This data can be found at: http://fcon_1000.projects.nitrc.org/indi/enhanced/access.html.

## Ethics Statement

The studies involving human participants were reviewed and approved by Institutional Review Board Approval was obtained for this project at the Nathan Kline Institute (Phase I #226781 and Phase II #239708) and at Montclair State University (Phase I #000983A and Phase II #000983B). Written informed consent was obtained for all study participants. Written consent and assent was also obtained from minor/child participants and their legal guardian. The patients/participants provided their written informed consent to participate in this study.

## Author Contributions

JS and DF conceptualized the study. JS conducted data analysis and drafted the manuscript. AB and DF contributed to the writing and editing of the manuscript, including suggestion of statistical approaches. All authors contributed to the article and approved the submitted version.

## Conflict of Interest

The authors declare that the research was conducted in the absence of any commercial or financial relationships that could be construed as a potential conflict of interest.
